# Long-Term Potentiation in Isolated Dendritic Spines

**DOI:** 10.1371/journal.pone.0006021

**Published:** 2009-06-23

**Authors:** Amadou T. Corera, Guy Doucet, Edward A. Fon

**Affiliations:** 1 Centre for Neuronal Survival and Department of Neurology and Neurosurgery, Montreal Neurological Institute, McGill University, Montreal, Quebec, Canada; 2 Groupe de Recherche sur le Système Nerveux Central and Département de Pathologie et de Biologie Cellulaire, Université de Montréal, Montréal, Québec, Canada; University of California, Berkeley, United States of America

## Abstract

**Background:**

In brain, *N*-methyl-D-aspartate (NMDA) receptor (NMDAR) activation can induce long-lasting changes in synaptic α-amino-3-hydroxy-5-methylisoxazole-4-propionate (AMPA) receptor (AMPAR) levels. These changes are believed to underlie the expression of several forms of synaptic plasticity, including long-term potentiation (LTP). Such plasticity is generally believed to reflect the regulated trafficking of AMPARs within dendritic spines. However, recent work suggests that the movement of molecules and organelles between the spine and the adjacent dendritic shaft can critically influence synaptic plasticity. To determine whether such movement is strictly required for plasticity, we have developed a novel system to examine AMPAR trafficking in brain synaptosomes, consisting of isolated and apposed pre- and postsynaptic elements.

**Methodology/Principal Findings:**

We report here that synaptosomes can undergo LTP-like plasticity in response to stimuli that mimic synaptic NMDAR activation. Indeed, KCl-evoked release of endogenous glutamate from presynaptic terminals, in the presence of the NMDAR co-agonist glycine, leads to a long-lasting increase in surface AMPAR levels, as measured by [^3^H]-AMPA binding; the increase is prevented by an NMDAR antagonist 2-amino-5-phosphonopentanoic acid (AP5). Importantly, we observe an increase in the levels of GluR1 and GluR2 AMPAR subunits in the postsynaptic density (PSD) fraction, without changes in total AMPAR levels, consistent with the trafficking of AMPARs from internal synaptosomal compartments into synaptic sites. This plasticity is reversible, as the application of AMPA after LTP depotentiates synaptosomes. Moreover, depotentiation requires proteasome-dependent protein degradation.

**Conclusions/Significance:**

Together, the results indicate that the minimal machinery required for LTP is present and functions locally within isolated dendritic spines.

## Introduction

Long-lasting, activity-dependent changes in synaptic function, such as those underlying long-term potentiation (LTP), are thought to represent the cellular basis for learning and memory [1]. Critical aspects of this plasticity are mediated by the *N*-methyl-D-aspartate (NMDA) and α-amino-3-hydroxy-5-methylisoxazole-4-propionate (AMPA) types of ionotropic glutamate receptors at excitatory synapses. To a large extent, activity-dependent stimulation of synaptic NMDA receptors (NMDARs) induces LTP by promoting trafficking of AMPA receptors (AMPARs) from endosomal pools within neurons into synaptic sites at the cell surface, which in turn strengthens synaptic transmission and mediates the expression of LTP [2]–[6]. Conversely, stimulation with AMPA induces a rapid internalization of surface AMPARs [7], [8].

AMPAR trafficking during LTP occurs within dendritic spines, protrusions connected to the dendritic shaft via a thin neck. However, recent work suggests that the movement of molecules and organelles between the spine and other neuronal compartments, such as the adjacent dendritic shaft, influences synaptic plasticity. Indeed, the spine neck dynamically regulates the diffusion of molecules between the spine and the rest of the neuron [9]. Moreover, endosomes appear to translocate from the dendritic shaft into the spine during LTP, possibly providing a supply of AMPARs for surface insertion and new membrane for spine expansion during plasticity [10], [11]. While these studies point to intricate regulatory mechanisms, they cannot definitively determine whether such movement is strictly required for plasticity, as they do not disrupt the connections between spines and adjacent compartments. Synaptosomes consist of presynaptic terminals attached to postsynaptic dendritic spines that have been disconnected from the adjoining dendritic shaft, suggesting they might serve as a model to study dendritic spines in isolation. Remarkably, key functions of intact neurons are conserved in synaptosomes, resulting in their well-established and extensive use for the study of synaptic functions such as neurotransmitter release and local protein synthesis [12], [13].

To determine whether dendritic spines can function autonomously in glutamatergic synaptic plasticity, we have developed a novel system to examine AMPAR trafficking in synaptosomes. We report here that synaptosomes can undergo LTP-like plasticity. Indeed, stimuli that mimic synaptic NMDAR activation, lead to a long-lasting increase in surface AMPAR levels, as measured by [^3^H]-AMPA binding and an increase in the levels of AMPAR subunits GluR1 and GluR2 in synapses. Importantly, these increases are not accompanied by changes in total AMPAR levels, consistent with the trafficking of AMPARs from internal synaptosomal compartments into synaptic sites. Moreover, this plasticity is reversible, as the application of AMPA after LTP depotentiates synaptosomes. The findings indicate that the minimal machinery required for LTP and AMPA-induced depotentiation is present and functions locally within dendritic spines.

## Materials and Methods

### Ethics Statement

All mouse experiments were performed under a protocol approved by the Montreal Neurological Institute Animal Care Committee in compliance with guidelines established by the Canadian Council on Animal Care.

### Drugs and Antibodies

AMPA, 2-amino-5-phosphonopentanoic acid (AP5), quisqualic acid, glycine, potassium thiocyanate (KSCN), Cycloheximide and protease inhibitors [benzamidine, aprotonin, leupeptin and phenylmethylsulfonylfluoride (PMSF)] were purchased from Sigma (St-Louis, MO). MG132 and lactacystin were from Calbiochem (San Diego, CA). Mouse monoclonal antibodies were used to detect GluR1 (Santa Cruz Biotechnology; Santa Cruz, CA), GluR2 (Chemicon; Temecula, CA), synaptophysin (Sigma; St-Louis, MO), PSD-95, NR2A, NR2B, CamKII, EEA1, Rab11, NR1 (BD Transduction Laboratories; San Jose, CA), and LAMP2 (Gift from Frederic Luton). Rabbit polyclonal antibodies were used to detect GluR2/3, GluR4 (Chemicon; Temecula, CA), and ERK (Santa Cruz Biotechnology; Santa Cruz, CA). Goat polyclonal antibodies were used to detect GRIP1, GRIP2 and PICK1 (Santa Cruz Biotechnology; Santa Cruz, CA).

### Preparation of Synaptosomes

Crude synaptosomes were prepared as described [14], [15]. Briefly, the brains of 1–2 C57BL/6 adult mice were homogenized in 10 volumes (w/vol.) of ice-cold solution A [0.32 M sucrose, 10 mM HEPES and protease inhibitors (100 µg/ml benzamidine, 0.5 µg/ml aprotonin, 0.5 µg/ml leupeptin and 20 µg/ml PMSF), pH 7.4]. All subsequent steps were carried out at 0–4°C. The nuclear material (P1) was removed by centrifugation at 1,000×g for 10 min. and the supernatant (S1) was centrifuged twice (12,000×g and 13,000×g) to obtain crude synaptosomes, which were re-suspended in physiological buffer [solution B consisting of (in mM): 125 NaCl, 26 NaHCO_3_, 1.6 NaH_2_PO_4_, 2.5 CaCl_2_, 5 KCl and 10 glucose, pH 7.4]. Synaptosomal protein concentrations were determined using bovine serum albumin as a standard [16].

### Transmission Electron Microscopic analysis of synaptosomes

Aliquots of synaptosomes (∼1 mg protein) were re-suspended in solution A, fixed with 2.5% glutaraldehyde in 0.2 M Sorensen's Phosphate Buffer (SPB) for 30 min. on ice and washed with 3 successive cycles of centrifugation (18,000×g). Synaptosomes were post-fixed with 2% osmium tetroxide in SBP for 30 min. on ice, washed 3 times with SPB and dehydrated in increasing concentration (50–100%) of ethanol followed by embedding in EPON 812 through successive incubations in increasing EPON 812/Propylene oxide ratios (1∶1 and 3∶1, respectively) and finally in “pure” EPON 812. Samples were cured at 58°C for 2 to 4 days. Ultrathin sections (∼10 nm) were then cut from the EPON blocks, transferred onto square-mesh copper grids and counter-stained with uranyl acetate and lead citrate. Micrographs were taken at 5,000–67,000× using a JEOL 100 CX electron microscope (JEOL, Peabody, MA, USA).

### Synaptic NMDA and AMPA Receptor Stimulation

For a typical synaptic NMDAR stimulation experiment, crude synaptosomes (∼400 µg) were pre-incubated with 100 µM glycine for 20 min. at 37°C in solution B, followed by the addition of either solution B alone (control) or solution B containing high KCl (50 mM final concentration) for various times (5 to 120 min.). For AMPAR stimulation, NMDAR stimulation was carried out as above for 10 min. followed by the addition of 100 µM AP5 to block NMDARs. Synaptosomes were then treated with either solution B alone (control) or with solution B containing 100 µM AMPA for different times. In certain experiments, synaptosomes were incubated with the indicated concentration of AP5, cycloheximide, MG-132 or lactacystin.

### Measurement of Surface and Total AMPA Receptor Levels using [^3^H]-AMPA

To determine surface AMPAR levels, treatment was stopped by addition of 1 ml of ice-cold binding buffer (100 mM Tris/Acetate, 0.1 mM EGTA, pH 7.4) followed by centrifugation (12,000×g/15 min. ×2). To determine total AMPAR levels, treatment was stopped by hypotonic lysis of synaptosomes in 1 ml of 20-fold diluted binding buffer containing protease inhibitors followed by a rapid sonication step (3×1 sec) and centrifugation (18,000×g) for 30 min. The resulting pellet (membranes) was re-suspended in binding buffer and centrifuged as above. In both cases, the final pellets (intact synaptosomes or lysed synaptosome membranes) were incubated for at least 30 min. on ice in 200 µl of binding buffer. [^3^H]-AMPA binding was carried out by incubating ∼100 µg aliquots for 60 min. in 100 µl of binding buffer containing 50 nM [^3^H]-AMPA (DL-α-[5-methyl- 3H], 40–70 Ci/mmol; Perkin Elmer, Boston, MA) and 50 mM KSCN on ice, as described [17]. Incubations were terminated by centrifugation at 18,000×g for 15 and 30 min. for intact synaptosomes and lysed synaptosome membranes, respectively. Pellets were rinsed once with 200 µl of binding buffer containing 50 mM KSCN prior to re-suspension in 100 µl of 0.2 N NaOH. The bound radioactivity was counted by liquid scintillation spectrometry with ∼50% efficiency. Specific [^3^H]-AMPA binding was determined by subtracting nonspecific (determined with 50 µM of the AMPAR antagonist quisqualic acid) from total binding. Saturation constants (K_d_ and B_max_) for [^3^H]-AMPA binding were determined with the nonlinear curve-fitting program Ligand software (Biosoft, Cambridge, UK), using a range of unlabelled AMPA [75–10000 nM] added to 50 nM of [^3^H]-AMPA.

### Preparation of postsynaptic density fractions

Fractions enriched in postsynaptic densities (PSDs) were prepared as described [14], [18]. Briefly, either treated or non-treated synaptosomes (∼3 mg of protein) were incubated in 50 mM Tris (pH 7.5) containing 0.5% Triton X-100 and protease inhibitors for 30 min. on ice followed by centrifugation at 32,000×g for 20 min. The PSD-enriched pellet was re-suspended in 50 mM Tris, pH 7.5. Aliquots from the synaptosome and PSD fractions (5 µg protein) were resolved by SDS-PAGE and immunoblotted with the indicated antibodies. The intensities of the bands of interest were determined using NIH imageJ.

### Statistical analysis

Specific binding of [^3^H]-AMPA to intact or lysed synaptosomes was expressed as femtomoles per milligram protein. Data are means±SEM of 3–4 independent experiments, carried out in duplicate. Student's t-tests were used to compare single treated versus control groups. Dunnett's t-test was used to compare several treated groups relatively to a reference group. ANOVAs followed by post-hoc analysis were used to compare multiple groups. p<0.05 was considered significant.

## Results

### Synaptic architecture is preserved in mouse brain synaptosomes

**Figure 1:**
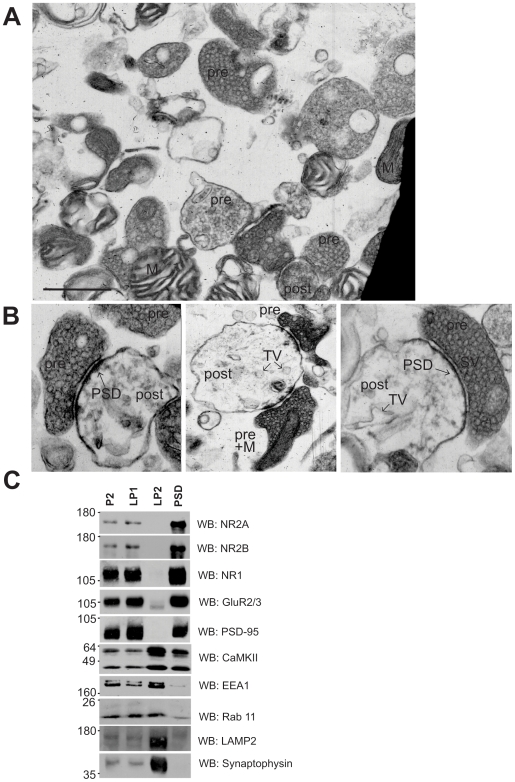
Ultrastructural and biochemical characterization of synaptosomes. A, Low power magnification of crude synaptosomes prepared from mouse brain showing pre-synaptic elements (pre) containing synaptic vesicles (SV) as well as post-synaptic elements (post), free mitochondria (M) and other unidentified structures. Scale bar corresponds to 0.5 µm. B, High power views of intact, sealed and tightly apposed pre- and post-synaptic elements, characteristic of asymmetric glutamatergic synapses. Clearly identifiable electron dense PSDs can be observed beneath the post-synaptic plasma membrane. The post-synaptic element also contains tubular and vesicular structures (TV) within the cytosolic compartment. Mitochondria could also be observed in the pre-synaptic terminal (pre+M). C, Fractionation of synaptosomes (P2) into synaptic plasma membrane- (LP1), synaptic vesicle- (LP2) and PSD-enriched fractions. Subcellular fractions were immunoblotted with antibodies against NMDA and AMPA receptor subunits (NR1 and GluR2, respectively) as well as markers of postsynaptic density (PSD-95), endosomal (EEA1, Rab11), lysosomal (LAMP2) and synaptic vesicle (Synaptophysin) compartments.

Using differential centrifugation, we isolated synaptosomes from mouse brain homogenates, as described [14], [15]. To determine whether synaptic architecture was preserved in our preparation, we used transmission electron microscopy to examine the synaptosomes at the ultrastructural level. Consistent with previous work [13], [19]–[21], we observed isolated membrane fragments and non-synaptic organelles, such as free mitochondria, in addition to intact synaptosomes (Fig. 1A). Importantly, we could easily identify synaptosomes in our preparation that consisted of intact and tightly apposed pre- and postsynaptic elements held in close register with each other (Fig. 1B). Presynaptic elements contained numerous, highly clustered clear synaptic vesicles as well as mitochondria. Further, a subset of synaptic vesicles appeared to be docked at the active zone, which was opposite obvious PSDs, electron-dense structures that function as synaptic signaling platforms containing NMDA and AMPA receptors as well as other signaling and scaffolding molecules [22], [23]. The postsynaptic elements, derived from pinched-off dendritic spines, also contained sparse membranous organelles, as observed in asymmetric excitatory synapses in tissue sections [11], [24]. Importantly, the appearance of the plasma membrane of the majority of spines appeared continuous suggesting that they were sealed and that their cytoplasmic contents were intact. Thin filaments could be observed in the synaptic cleft, consistent with the preservation of transynaptic adhesion molecules between the pre- and postsynaptic structures [20]. Taken together, these morphological characteristics ascertain that our purification procedure did not disrupt the structural integrity or general architecture of synapses in our synaptosome preparation, and thereby fulfill an important prerequisite for their use in the study of synaptic AMPAR trafficking.

### Biochemical characterization of synaptosomes

In order to determine the distribution of proteins implicated in glutamate synaptic transmission and plasticity within our preparation, we further fractionated synaptosomes (P2) into synaptic plasma membrane- (LP1), synaptic vesicle- (LP2) and PSD-enriched fractions (Fig. 1C). Consistent with previous work [21], we found that the AMPAR subunits GluR2/3 were present in synaptosomes and distributed in the LP1 and PSD fractions, and to a lesser extent the LP2 fraction. The NMDAR subunits NR1, NR2A, NR2B and the major postsynaptic scaffolding protein PSD-95 were also distributed in the LP1 and PSD fractions but were not detected in LP2. In contrast, Calcium/calmodulin-dependent protein kinase II (CaMKII), a major synaptic kinase, was enriched in both LP2 and PSD fractions whereas synaptophysin, a major synaptic vesicle protein was enriched in the LP2 fraction, as expected. Interestingly, we also found that Early Endosome Antigen 1 (EEA1), Rab11 and LAMP2 were present in synaptosomes and enriched in the LP2 fraction. Although traditionally associated with synaptic vesicles, the LP2 fraction also contains other vesicle populations [21]. Our finding that the endosomal proteins EEA1 and Rab11 and the lysosomal protein LAMP2 were enriched in this fraction suggests, that at least a subset of vesicles in LP2 are endosomes and lysosomes. Thus, consistent with the ultrastructural data above, the fractionation procedure confirms that key proteins involved in synaptic plasticity and trafficking are not only present but also localized to appropriate subcellular compartments within synaptosomes.

### Synaptic NMDA receptor stimulation increases surface AMPA receptors in synaptosomes

**Figure 2:**
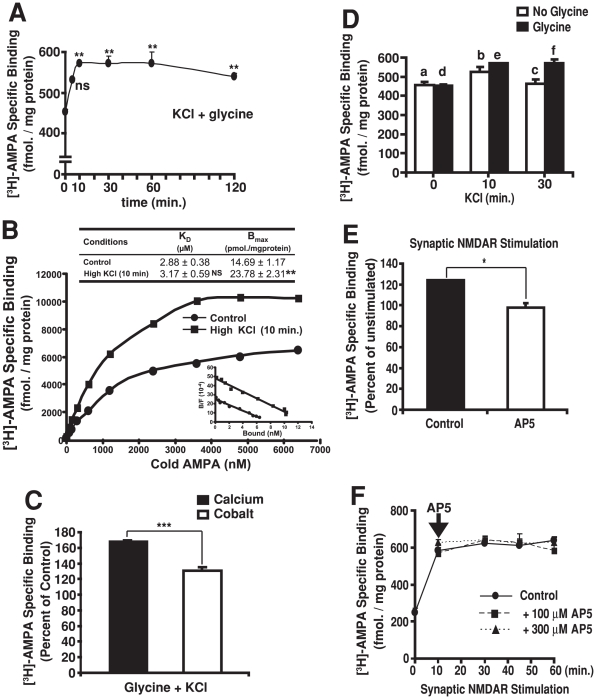
Synaptic NMDAR stimulation induces LTP in synaptosomes. A, Sustained increase in surface AMPAR levels in synaptosomes following KCl and glycine stimulation. Synaptosomes were incubated at 37°C for 20 min. in the presence of glycine (100 µM) followed by depolarization with high KCl (50 mM) for 5–120 min. B, Saturation curves and Scatchard plots (inset) of [^3^H]-AMPA binding to non-permeabilized control and stimulated synaptosomes. The K_d_ and B_max_ are shown in the table above. C, Increase in surface AMPAR levels is calcium-dependent. Synaptosomes were incubated in physiological buffer containing 2.5 mM of either calcium or cobalt and stimulated with high KCl for 30 min. as in A. D, Stimulation with the NMDAR co-agonist glycine is required for a sustained increase in surface AMPAR levels. Synaptosomes were incubated in the presence or absence of glycine (100 µM) and subsequently stimulated with high KCl as in A. E, The NMDAR antagonist AP5 prevents the glycine + KCl-induced increase in surface AMPAR levels. Synaptosomes were pre-treated either with or without AP5 (100 µM) and stimulated for 10 min. as in A. F, Blockade of NMDARs after initiating glycine + KCl stimulation does not block the increase in surface AMPAR levels. Synaptosomes were stimulated as above, followed by the addition (arrow) of either physiological buffer (control) or AP5 at the indicated concentrations. For all experiments, surface AMPAR levels were determined by measuring the specific binding of [^3^H]-AMPA to non-permeabilized synaptosomes and values represent means±SEM of 3 to 5 independent experiments. Dunnett's test was used to compare KCl-treated vs. control in A; **, p<0.01; ns, non significant. Student's t-test was used in C and E; *, p<0.05; ***, p<0.001. Two-way ANOVA followed by Holm-Sidak method was used for pair-wise comparisons in D between a and b, p<0.05; a and c, non significant; d and e, p<0.05; d and f, p<0.05. Groups were compared using one-way ANOVA in F; F(2,44) = 0.0587; p = 0.943, not significant.

In cultured hippocampal neurons, application of glycine, an NMDAR co-agonist, results in the activation of synaptic NMDARs by endogenously released glutamate [2], [25]. This selective stimulation of synaptic NMDARs induces LTP of AMPAR-mediated miniature excitatory postsynaptic currents (mEPSCs) and is accompanied by a rapid surface insertion of AMPARs into synaptic sites. Using a similar strategy, we devised a method to activate NMDARs by the release of endogenous glutamate from presynaptic terminals in synaptosomes. First, we incubated synaptosomes in a solution containing a saturating concentration of the NMDAR co-agonist glycine (100 µM). Next, we depolarized the synaptosomes using a high concentration of KCl (50 mM), in the presence of a physiological concentration of Ca^2+^ (2.5 mM). As this induces the exocytosis of synaptic vesicles and release of endogenous glutamate from presynaptic terminals, it preferentially activates synaptic NMDARs, which are located at the PSD, directly across from the presynaptic active zone. In order to determine the levels of assembled multimeric AMPARs expressed at the surface of synaptosomes, we measured the specific binding of [^3^H]-AMPA to non-permeabilized synaptosomes. We observed a rapid increase in surface [^3^H]-AMPA binding that persisted for the duration of the experiment (Fig. 2A). Moreover, both the kinetics and the magnitude of the increase are virtually identical to the increase in mEPSCs observed during LTP in cultured neurons [25]. To determine whether the increase in [^3^H]-AMPA binding to synaptosomes reflected an increase in the number of surface AMPA binding sites or an increase in the affinity of receptors for AMPA, we carried out saturation binding studies. We observed a significant increase in B_max_ but not K_d_ values after stimulation (Fig. 2B), consistent with an increase in the number of AMPARs at the surface without change in receptor affinity. Ca^2+^ is required for synaptic vesicle exocytosis leading to evoked presynaptic glutamate release and for postsynaptic signaling via the NMDAR during LTP. Indeed, substitution of Ca^2+^ with Co^2+^ in the incubation buffer attenuated the increase in surface AMPAR levels (Fig. 2C). Further, stimulation with KCl in the absence of glycine resulted in only a transient increase in surface [^3^H]-AMPA binding at 10 minutes followed by a decrease to baseline at 30 minutes (Fig. 2D). Similarly, treatment with glycine alone did not increase surface AMPAR levels (data not shown). Together, the findings indicate that both depolarization with KCl to release endogenous glutamate from presynaptic terminals and co-activation of NMDARs with glycine are required to induce a sustained increase in surface AMPARs in synaptosomes. Next, to examine the role of synaptic NMDAR stimulation more specifically, we used AP5, a competitive NMDAR antagonist. Pre-incubation of synaptosomes with AP5 blocked the increase in surface AMPAR levels (Fig. 2E). In contrast, the addition of AP5 at later time points, after the initial increase in surface AMPAR levels, did not reduce levels back down to baseline (Fig. 2F). Further, treatment with AP5 and glycine without depolarization with KCl had no effect on surface AMPAR levels (data not shown). Together, these observations indicate that the activation of synaptic NMDARs is required for the induction of LTP-like plasticity in synaptosomes, as is the case in brain slices and cultured neurons.

### Synaptic NMDA receptor stimulation redistributes AMPA receptors into synapses

**Figure 3:**
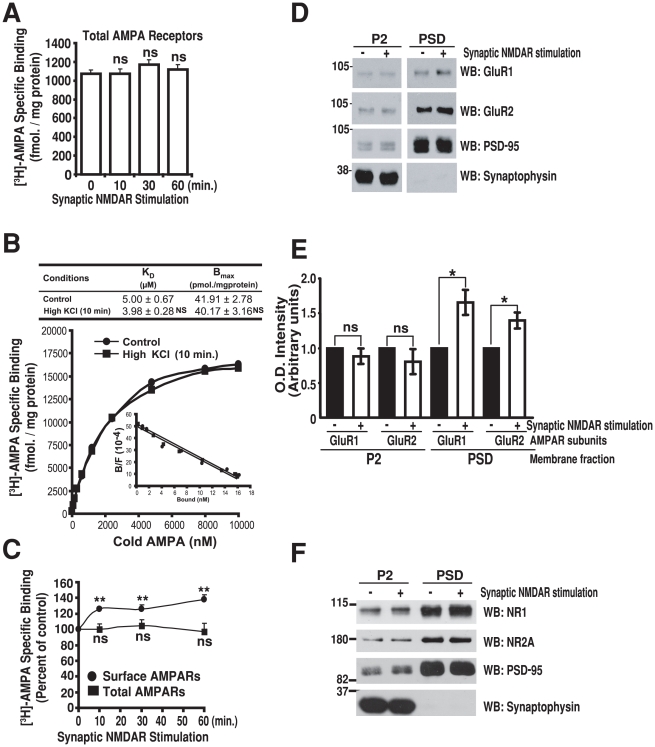
LTP in synaptosomes promotes the translocation of AMPARs into synaptic sites. A, Synaptic NMDAR stimulation does not change total AMPAR levels. Synaptosomes were submitted to synaptic NMDAR stimulation as in figure 2, lysed hypotonically and sonicated, followed by [^3^H]-AMPA binding to determine total AMPAR levels. B, Saturation curves and Scatchard plots (inset) of total [^3^H]-AMPA binding in control and stimulated synaptosomes. The K_d_ and B_max_ are shown in the table above. C, Synaptic NMDAR stimulation translocates AMPARs from intra-synaptosomal compartments to the surface. Synaptosomes were treated as in A and both surface and total AMPAR levels determined. D and E, Synaptic NMDAR stimulation leads to the insertion of AMPARs into PSDs. Synaptosomes were either left untreated or stimulated as above for 10 min. and PSD fractions were purified. Both synaptosome and PSD fractions were immunoblotted with indicated antibodies (D) and O.D. intensities were determined (E). F, Synaptic NMDAR stimulation does not change NMDAR subunit levels in PSDs. Synaptosome were treated and PSD fractions were prepared as in (D) and immunoblotted with the indicated antibodies. For all experiments, values are means±SEM of 3 to 5 independent experiments. Dunnett's test was used to compare stimulated vs. control synaptosomes in A and B; **, p<0.01; ns, non significant. Student's t-test was used to compare treated vs. untreated synaptosomes in D; *, p<0.05; ns, non significant.

The increase in surface AMPAR levels could be explained by an increase in the total number of AMPARs in synaptosomes, for instance via local synthesis of new receptors. Alternatively, existing AMPARs could be redistributed from internal membrane compartments to the surface of synaptosomes. To distinguish between these possibilities, we determined total AMPAR levels by disrupting synaptosomes using hypotonic lysis and sonication, followed by [^3^H]-AMPA binding to both internal and surface pools of AMPARs. Interestingly, total AMPAR levels did not change after synaptic NMDAR stimulation (Fig. 3A). Similarly, saturation binding studies did not show significant differences between baseline and stimulated K_d_ and B_max_ values for [^3^H]-AMPA binding to total synaptosome membranes (Fig. 3B), indicating that the observed increase in surface AMPAR levels (Fig. 2A, B) resulted from a redistribution of existing AMPARs from internal compartments to the surface of synaptosomes rather than from an increase in the total levels of AMPARs (Fig. 3C). Indeed, extrapolating from B_max_ values for surface and total [^3^H]-AMPA binding, in figures 2B and 3B respectively, we estimate that the proportion of all AMPARs expressed on the surface of synaptosomes increases from ∼35% to ∼59% after synaptic NMDAR stimulation. Together, these findings are in line with previous work in cultured neurons, indicating that a large proportion of AMPARs reside in intracellular pools that can be rapidly translocated to the cell surface in response to stimuli that induce LTP [2], [25].

Binding of [^3^H]-AMPA, to non-permeabilized synaptosomes, measures all surface AMPAR levels, which includes both synaptic and extra-synaptic receptors. Synaptic glutamate receptors are clustered at the PSD, which contains detergent-insoluble receptor signaling protein complexes involved in glutamate neurotransmission and plasticity [22], [23], [26]. To determine whether the increase in surface AMPAR levels corresponds to an increase in synaptic receptors, we purified the Triton X-100-insoluble PSD fraction from synaptosomes, as described previously [14], [18], and used immunoblotting to determine the levels of the AMPAR subunits GluR1 and GluR2 (Fig. 3D). We found that synaptic NMDAR stimulation increased both GluR1 and GluR2 levels within the PSD fraction (Fig. 3E). In contrast, GluR1 and GluR2 levels in the total synaptosome fraction did not change after stimulation, consistent with the lack of change in total AMPAR levels described above (Fig. 3A, 3B). The increase in synaptic AMPARs was specific, as synaptosome stimulation did not change the levels of the NMDAR subunits NR1 and NR2A in the PSD (Fig. 3F) nor did it affect the levels of PSD-95, a protein enriched in PSD fractions. Further, synaptophysin, a presynaptic synaptic vesicle marker, was absent from our PSD fractions, attesting to the purity of our PSD preparation. Taken together, our data indicate that synaptic NMDAR stimulation results in a rapid, specific and sustained increase in synaptic AMPARs in synaptosomes, as is the case in cultured neurons. Considering that the insertion of additional AMPARs into synaptic sites is believed to underlie the expression of LTP, our results demonstrate that fundamental features of LTP can be reconstituted in synapses isolated from their dendritic shafts, axons and cell bodies.

### AMPA receptor stimulation reduces surface AMPA receptor levels in synaptosomes

**Figure 4:**
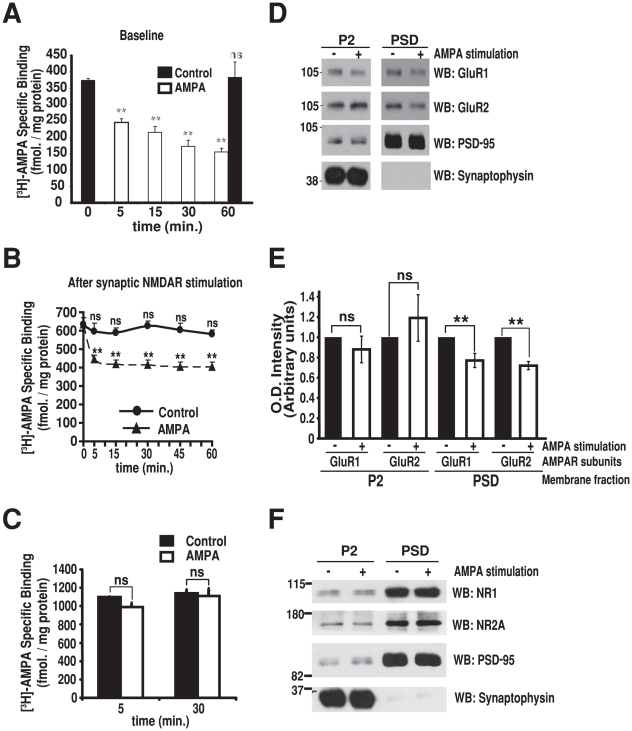
AMPA depotentiates synaptosomes. A, Synaptosomes were either left untreated (0 and 60 min. time points) or treated with AMPA (100 µM) for the indicated times. B, LTP was induced in synaptosomes and AP5 (100 µM) was added as in figure 2F. After 10 min., samples were either left untreated (0 time point) or treated with AMPA (100 µM) for the indicated times. C, AMPA stimulation after LTP does not change total AMPAR levels. Synaptosomes were treated as in B and then lysed hypotonically and sonicated, followed by [^3^H]-AMPA binding to determine total AMPAR levels. The decrease in surface AMPARs (B) without changing total AMPAR levels (C) indicates that AMPARs are internalized after AMPA treatment in synaptosomes. D and E, AMPA stimulation leads to the removal of AMPAR from PSDs. Synaptosomes were either left untreated or treated as in B for 10 min. and PSD fractions were purified. Both synaptosome and PSD fractions were immunoblotted with indicated antibodies (D), and O.D. intensities were determined (E). F, AMPA stimulation after LTP does not change NMDAR subunit levels in PSDs. Synaptosome were treated and PSD fractions were prepared as in (D) and immunoblotted with the indicated antibodies. For all experiments, AMPAR levels were determined by measuring specific binding of [^3^H]-AMPA and values are means±SEM of 3–4 independent experiments. One-way ANOVA on AMPA effect followed by Fisher-Snedecor F test was used in A; F(5,12) = 17.89 ; **, p<0.001 Dunnett's t-test. Two-way ANOVA followed by Bonferroni t-test was used in B; AMPA, F(1,47) = 55.43, p<0.001; Time, F(5,36) = 4.42, p<0.05; AMPA x Time, F(5,47) = 2.413, not significant; ***, p<0.001; ns, not significant. Student's t-test was used in C and E; **, p<0.01; ns, non significant.

Work in cultured neurons has shown that application of AMPA results in a robust internalization of AMPARs from the cell surface [7]. Using a similar paradigm in synaptosomes, we found that the application of AMPA (100 µM), either at baseline (Fig. 4A) or 10 minutes after the initial NMDAR stimulation phase (Fig. 4B), resulted in a rapid and sustained decrease in surface [^3^H]-AMPA binding, consistent with ligand-induced AMPAR internalization. In the latter case (Fig. 4B), the effect was observed only when AP5 was co-administered with AMPA in order to block ongoing NMDAR stimulation (data not shown). Again, this is consistent with work in cultured neurons showing that, in contrast to the isolated stimulation of AMPARs, application of NMDA promotes the rapid surface reinsertion of AMPARs after internalization [7]. Further, the levels of surface AMPARs after application of AMPA (Fig. 4B) were similar to levels prior to the initial synaptic NMDA stimulation (Fig. 2A) but did not decrease below this level. Thus, AMPA-induced receptor internalization depotentiates synaptosomes after LTP and attests to the resilience of our preparation for the study of bi-directional AMPAR trafficking.

### AMPA receptor stimulation after LTP redistributes AMPA receptors away from synapses

The observed AMPA-induced decrease in surface AMPAR levels after LTP can be explained either by a decrease in the total number of AMPARs in synaptosomes or by a redistribution of existing AMPARs from the surface of synaptosomes to internal compartments. We found that application of AMPA for 30 minutes did not decrease total AMPAR levels in permeabilized synaptosomes, indicating that the decrease in surface AMPAR levels, results from a redistribution of AMPARs from the surface to internal synaptosomal compartments (Fig. 4C). To determine whether the decrease in surface AMPAR levels corresponds to a removal of receptors from synaptic sites, we purified PSD fraction from the synaptosomes, as above, and used immunoblotting to determine the levels of the AMPAR subunits GluR1 and GluR2 within the PSD fractions (Fig. 4D). We found that AMPAR stimulation decreased both GluR1 and GluR2 levels within the PSD fraction (Fig. 4E). In contrast, GluR1 and GluR2 levels in the total synaptosome fraction did not change after stimulation, consistent with the lack of change in total AMPAR levels described above (Fig. 4C). Further, the ligand-induced decrease in synaptic AMPARs was specific, as synaptosome stimulation did not change the levels of the NMDAR subunits NR1 and NR2A in the PSD (Fig. 4F) nor did it affect the levels of PSD-95 or synaptophysin. Thus, AMPA-induced depotentiation of synaptosomes after LTP is accompanied by a rapid and specific removal of AMPAR subunits from synaptic sites.

### LTP in synaptosomes does not require protein synthesis or proteasome-dependent protein degradation

**Figure 5:**
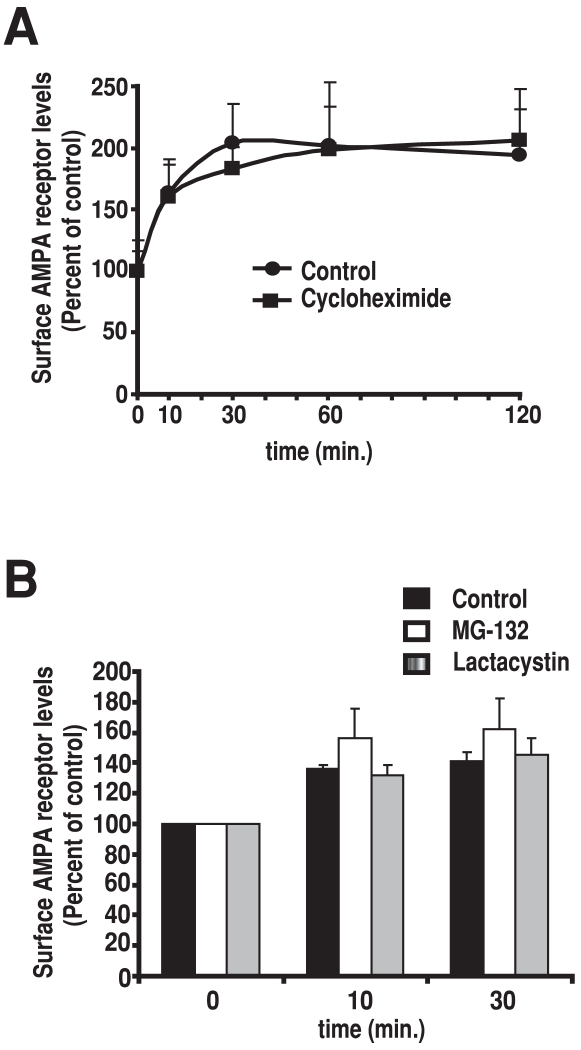
LTP in synaptosomes does not require new protein synthesis or proteasome-dependent protein degradation. A, Inhibition of new protein synthesis with cycloheximide does not inhibit LTP in synaptosomes. Synaptosomes were pre-treated either with or without cycloheximide (50 µM) and stimulated as in figure 2A. No significant differences in surface AMPAR levels were found between cycloheximide-treated and control synaptosomes. B, Inhibition proteasome-dependent protein degradation does not inhibit LTP in synaptosomes. Synaptosomes were pre-treated with either the proteasome inhibitor MG-132 (50 µM), lactacystin (10 µM) or control buffer and stimulated as in figure 2A. No significant differences in surface AMPAR levels were found between either proteasome inhibitor and control synaptosomes. In both (A) and (B), surface AMPAR levels were determined by measuring specific binding of [^3^H]-AMPA to non-permeabilized synaptosomes and values represent means±SEM of 3 independent experiments.

Synaptosomes and synaptoneurosomes have long been used as model systems to study synaptic protein synthesis [13]. Moreover, local protein synthesis has been implicated in LTP in hippocampal slices, cultured neurons and *in vivo* [27]–[31]. We therefore pre-incubated synaptosomes with the protein synthesis inhibitor cycloheximide (50 µM) prior to stimulation with KCl and glycine. We found no difference in the magnitude or in the duration of increase in surface AMPARs after synaptic NMDAR stimulation between control and cycloheximide-treated synaptosomes (Fig. 5A). New protein synthesis is particularly important for the maintenance of LTP but is not believed to be required for the induction of LTP [27], [29], [31]. Consistent with this notion, cycloheximide did not block the induction of LTP in synaptosome. Moreover, the increase in surface AMPARs persisted for 2 hours, suggesting that new protein synthesis is not required for maintenance of LTP over this time period in our system.

The ubiquitin-proteasome system is the major pathway for protein degradation in cells. Proteasome-dependent protein degradation regulates various aspects of neuronal function, including synaptic plasticity [32]–[35]. We therefore pre-incubated synaptosomes with the proteasome inhibitors MG-132 (50 µM) or lactacystin (10 µM) prior to stimulation with KCl and glycine. As was the case with cycloheximide, neither MG-132 nor lactacystin blocked the increase in surface AMPARs after synaptic NMDAR stimulation (Fig. 5B), consistent with a recent report showing that early LTP was insensitive to proteasome inhibitors in brain slices preparations [29]. Together, our findings indicate that LTP in isolated dendritic spines occurs independently of protein synthesis and degradation.

### AMPA-induced depotentiation in synaptosomes requires proteasome function

**Figure 6:**
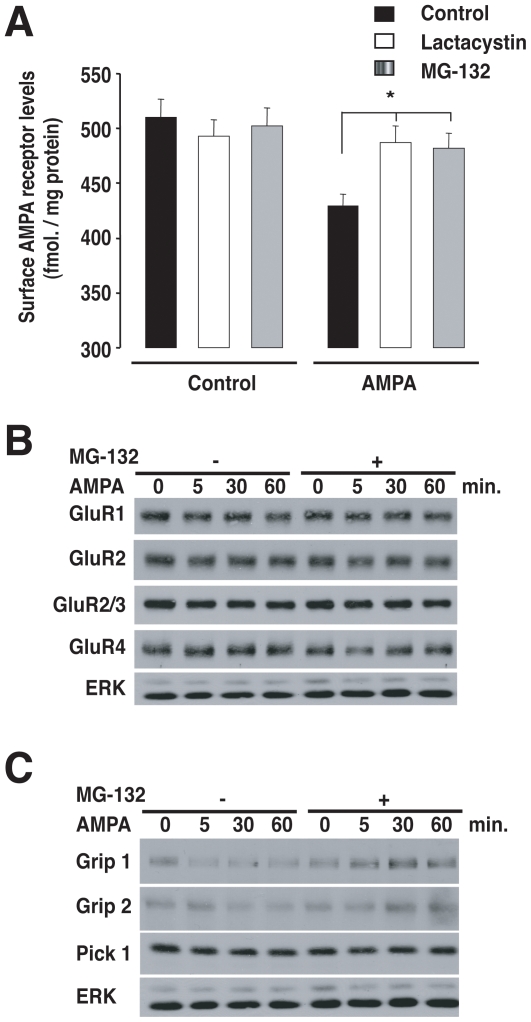
Proteasome function is required for AMPA-induced depotentiation of synaptosomes. A, Proteasome inhibitors block AMPA-induced AMPAR internalization after LTP in synaptosomes. LTP was induced in synaptosomes and AP5 (100 µM) was added as in figure 2F. After 10 min., samples were treated with either the proteasome inhibitor MG-132 (50 µM), lactacystin (10 µM) or control buffer, followed by AMPA (100 µM) for 30 min. to depotentiate synaptosomes as in figure 4B. Both proteasome inhibitors completely blocked the AMPA-induced reduction surface AMPAR levels, as determined by specific binding of [^3^H]-AMPA to non-permeabilized synaptosomes. Values represent means±SEM of 3 independent experiments. Dunnett's test was used to compare proteasome inhibitor vs. control synaptosomes. *, p<0.05. B and C, AMPA-induced depotentiation of synaptosomes leads to the proteasome-dependent degradation of AMPAR scaffolding protein (C) but not AMPAR subunits (B). Synaptosomes were depotentiated after LTP by incubating with AMPA (100 µM) for the indicated times as in figure 4B. The samples were then immunoblotted with indicated antibodies against the GluR AMPAR subunits (B) or against the AMPAR adaptor proteins GRIP1, GRIP2 and PICK1 (C). Anti-Erk was used as a loading control. Only GRIP1 and GRIP2 levels decreased in response to AMPA-induced depotentiation. The decrease was blocked by MG-132.

**Figure 7:**
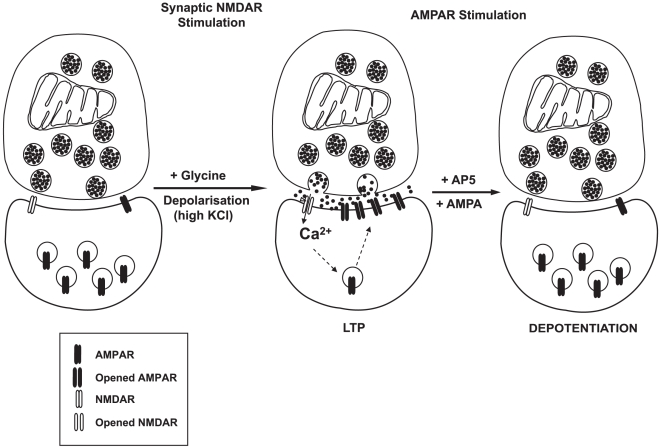
Model of LTP and AMPA-mediated depotentiation in synaptosomes. *Synaptic NMDAR stimulation*, High KCl concentration depolarizes synaptosomes releasing endogenous glutamate, which activates synaptic NMDARs in conjunction with the NMDAR co-agonist glycine. Both glutamate and glycine cooperate to open receptor channels, facilitating calcium influx into synaptosomes, which, in turn, initiates a cascade of events resulting in the translocation of AMPARs from internal pools into synaptic sites. *AMPAR stimulation*, After LTP, AMPA depotentiates synaptosomes by translocating AMPARs from synapses into internal pools.

AMPAR internalization has been shown to be sensitive to proteasome inhibitors in cultured neurons [36], [37]. Thus, we asked whether proteasome-dependent protein degradation could regulate AMPA-induced depotentiation after LTP in synaptosomes. Synaptosomes were stimulated with KCl and glycine for 10 minutes to increase surface AMPARs, followed by incubation with AMPA as above (Fig. 4B). The decrease in surface AMPAR levels was completely blocked by incubation with either lactacystin or MG132 (Fig. 6A). Thus, agonist-induced depotentiation in isolated dendritic spines requires proteasome function. Next, we asked whether the AMPARs *per se* were targets of proteasomal degradation. As shown in Figure 4C, total AMPAR levels, as measured by [^3^H]-AMPA binding in lysed synaptosomes, do not decrease after 30 minutes of AMPA-induced depotentiation. We extended this time course to 1 hour and examined the effects of proteasome inhibitors on the levels of individual AMPAR subunits by immunoblotting (Fig. 6B). We found no change in the levels of GluR1, GluR2, GluR2/3 and GlurR4 in synaptosome treated for up to 1 hour with AMPA, either with or without proteasome inhibitors. The results suggest that AMPA-induced depotentiation after LTP does not operate by direct degradation of AMPARs. Next, we asked whether adaptor proteins, which are key mediators of AMPAR trafficking, such the PDZ proteins GRIP1, GRIP2 and PICK1 could be targets of the proteasome [38]. We found that both GRIP1 and GRIP2, but not PICK1 levels, decreased upon AMPA-induced depotentiation in synaptosomes (Fig. 6C). Importantly, the decrease involved proteasome-mediated degradation of GRIP1 and GRIP2, as it could be blocked by MG-132. The findings are consistent with recent work in cultured neurons, indicating that GRIP1 is targeted to the proteasome in response to glutamate stimulation [37]. Thus, our findings in synaptosomes indicate that AMPA-induced depotentiation after LTP requires proteasome function, which targets adaptor proteins such as GRIP1 and GRIP2 rather than the AMPAR themselves for degradation. Taken together, both the pattern of changes and the regulatory mechanisms involved strongly suggest that key features of both NMDA- and AMPA-dependent AMPAR trafficking are regulated locally and can be reconstituted in individual synaptic units consisting of isolated presynaptic terminals and dendritic spines (Fig. 7).

## Discussion

In this study, we have established that key features of LTP and ligand-induced AMPAR internalization can be reconstituted in mouse brain synaptosomes. The major finding is that isolated mammalian synapses, severed from their adjoining axons and dendritic shafts and disconnected from the more distant cell bodies, are not only competent to carry out the functions associated with basic neurotransmission but can also robustly emulate core adaptations associated with glutamatergic synaptic plasticity. Moreover, as synaptosomes yield large quantities of protein, our system may provide a unique tool for the biochemical analysis of LTP compared to more traditional approaches, which are better suited for electrophysiological and imaging studies. The findings imply that at least the minimal synaptic signaling machinery associated with LTP is present in our synaptosome preparation. Indeed, we find that the synaptosomes express the AMPAR subunits GluR1-4, which are the major transducers of synaptic activity at glutamate synapses and are required for the expression of LTP. Moreover, the receptors are likely to be functional, as single channel recordings have been carried out recently from synaptosomal AMPARs [39]. Consistent with previous studies, we find that other critical mediators of synaptic plasticity such as NMDARs and PSD-95 are also present in our preparation [40], [41]. However, the mere presence of essential signaling molecules does not guarantee that their functions will be orchestrated to generate LTP unless they are organized in the correct configuration within structurally intact synapses.

At the ultrastructural level, our preparation yielded easily identifiable synaptic profiles consisting of tightly apposed and sealed pre- and postsynaptic elements with morphological characteristics of asymmetric excitatory synapses, as described previously [19], [21]. The presynaptic terminals contained synaptic vesicles clustered at active zones directly across from characteristic PSDs in dendritic spines. A sizeable fraction of AMPARs were present at the surface of synaptosomes and co-fractionated with NMDARs and PSD-95, indicating they were localized to synapses. We also observed vesicular and tubular structures within postsynaptic elements. These are likely to be endosomes, which have been described within dendritic spines [11], [21], [24]. Indeed, we found that a substantial pool of AMPARs were localized to internal compartments within synaptosomes and co-fractionated with the endosomal markers Rab11 and EEA1. Thus it appears that our synaptosomes not only express key proteins involved in plasticity but also display intact synapses containing AMPARs appropriately localized to subcellular compartments poised to carry out the trafficking events associated with LTP and depotentiation.

An increase in surface AMPARs at synaptic sites is believed to underlie the enhanced synaptic strength associated with LTP. Importantly, the increase in surface AMPAR levels we observe in synaptosomes closely mimics properties of LTP observed in more traditional systems. For instance, the increase was induced by the release of endogenous glutamate from presynaptic sites. It is well established that depolarization of synaptosomes with KCl evokes Ca^2+^-dependent exocytosis of synaptic vesicles and release of endogenous neurotransmitter from presynaptic terminals [12]. Moreover, LTP is typically triggered by the stimulation of synaptic NMDARs. The preserved synaptic architecture we observed by EM suggests synaptosomes are competent, not only for neurotransmitter release but also for synaptic transmission, with synaptic glutamate receptors directly across from the site of release at presynaptic active zone being preferentially activated. The effect is likely to be NMDAR-dependent as it required the application of the NMDAR co-agonist glycine and could be blocked by the NMDAR antagonist AP5. Further, both the time scale and the magnitude of the increase were virtually indistinguishable from those observed in LTP in cultured neurons [25]. Moreover, the increase in surface AMPA binding correlates with an increase in the amounts of GluR subunits localized to the PSD fraction, without changing the total abundance of GluRs. Taken together, the findings suggest that regulated AMPAR trafficking and insertion into synaptic sites, in response to stimuli that induce LTP, can be efficiently reconstituted in synaptosomes and likely reflect the mechanisms that underlie LTP in more traditional systems.

Synaptosomes have been used previously to examine surface AMPA binding. Whereas increases have been noted after depolarization with KCl, the lack of glycine during stimulation and the lack of inhibition by NMDA antagonists, as well as other methodological differences suggest it was unlikely to correspond to LTP [42]. More recently, decreases in surface biotin-labeled postsynaptic GluR subunits were detected in synaptosomes after AMPA and NMDA stimulation [43]. We also found that stimulation with AMPA decreases surface AMPAR levels. However, significant differences between the fractionation, stimulation and receptor quantification methodologies make it difficult to compare the two approaches directly. For instance, we applied AMPA to synaptosomes after the initial induction of LTP. Our paradigm is therefore more in keeping with AMPA-induced depotentiation. Indeed, we found that AMPA stimulation provoked a decrease in surface receptor levels back down to those observed before the induction of LTP but not below this baseline. The reduction in surface AMPARs likely corresponds to their removal from synaptic sites, as it correlates with a reduction in GluR levels in the PSD without changes in total GluR levels. Maybe most importantly, as the initial increase in surface AMPA binding is reversible, it is unlikely to be due to damage, rupture or permeabilization of synaptosomes during the stimulation protocol required to induce LTP. Interestingly, we found that AMPAR internalization required ubiquitin-proteasome function, consistent with previous work [36]. The process did not involve degradation of AMPAR *per se*. Instead, the PDZ-domain AMPAR adaptor proteins GRIP1 and GRIP2, but not PICK1, were targeted for degradation. GRIP degradation in synaptosomes was induced by AMPA stimulation whereas similar findings in cultured neurons were reported to be NMDA-dependent [37]. Despite theses differences, a role for stimulation-induced degradation of GRIPs in AMPAR internalization fits well with their proposed function in stabilizing AMPARs at the surface [44], [45]. Taken together, the ability to depotentiate synaptosomes after LTP and the shared mechanisms with more conventional systems attest to their resilience and further validates their use for the study of synaptic plasticity.

A large body of work points to the dendritic spine as the principal signaling hub responsible for transducing excitatory glutamatergic synaptic transmission and for the expression of postsynaptic plasticity, including most forms of LTP. Recent work, using two-photon uncaging of glutamate, has made it possible to stimulate and resolve events occurring at the level of individual synaptic spines [46]–[48]. Despite these advances, the precise subcellular structures involved in the induction and expression of LTP have been difficult to delimit with current approaches because experiments in brain slices and cultured neurons cannot definitively localize these processes, as they do not disrupt the connections between spines and adjacent structures. Moreover, experiments transecting dendrites in cultured neurons do not have the resolution to physically separate dendritic shafts from spines [49]. In contrast, the postsynaptic elements in our synaptosomes are pinched off from the adjacent dendritic shaft, thereby providing a means to study plasticity in physically isolated dendritic spines. This may be relevant as recent work implicates the spine neck, located at the junction between the dendritic shaft and the spine head, as an important point of regulation in synaptic plasticity. Indeed, the spine neck appears to filter membrane potentials and dynamically regulates the diffusion of molecules between the spine and the rest of the neuron, suggesting that at least under certain circumstances spines may be functionally isolated [9], [50], [51]. Plasticity induces important structural changes in dendritic spines, including a transient expansion of spines during LTP [47], [52], [53]. Recent work implicates endosomes as a source of new membrane lipids for structural spine expansion [11], in addition to providing a supply of AMPARs for surface insertion during LTP [2]. In a process involving the actin-based motor myosin V and the small GTPases Rab11, endosomes have been shown to translocate from the dendritic shaft at the base of the spine into the spine head during LTP [54], [55]. Whereas such translocation undoubtedly regulates critical aspects of plasticity, our work indicates that movement of molecules and membranes between the dendritic shaft and the spine is not absolutely required for LTP. Rather, it appears that the subpopulation of Rab11-positive endosomes, already present within pinched-off spines in synaptosomes (Fig. 1), suffice to mediate plasticity. While we do not claim that this reductionist approach in a simple system can capture all aspects of the intricate regulation of AMPAR plasticity, our findings imply that at least the minimal machinery required for LTP resides and functions locally within dendritic spines. Given that key features of glutamate receptor signaling and trafficking can be reconstituted in synaptosomes, our work unequivocally assigns the locus of LTP to the dendritic spine. Moreover, the large protein yields and ease of preparation make synaptosomes a unique and convenient tool for future proteomic studies of LTP and other forms of synaptic plasticity.
